# The Cholera

**Published:** 1854-11

**Authors:** 


*The Cholera.*—Setting all minor (and their name is legion) methods aside, we may draw attention to the following chief practices that have been "trumpet-tongued" advised in the treatment of cholera since its first appearance in 1831–32; each one for the time being lauded to the skies, and each one in succession bowing to its more fashionable and novel successor.

First.—There was the stimulating plan, according to which alcoholic compounds, etherial fluids, ammonia, volatile oils, pepper, spices, nux vomica, and camphor, were the drugs whose aid was mainly to be sought.

Secondly.—There was the Indian plan, as some called it, in which one scruple or half-drachm doses of calomel were given for a few times, and then followed at (variable) intervals, by ten, five, two grains in succession.

Thirdly.—There was the calomel and opium method, according to which it was conceived that opium, whether in combination with large or small doses of mercury, or anything else, played a most essential and indeed unequalled part; modifications of which opium and calomel plan were innumerable, but those in which this combination was accompanied by venesection on the one hand, and by the warm or vapor bath on the other, were perhaps the more important.

Fifthly,—Arose the saline plan of treatment, in which one party advised the administration of large and frequent doses of certain salts of potash and soda by mouth, while another proposed and practised the injection of them directly into the venous system.

Sixthly.—Very great stress was laid, and still is by many, upon that view of the therapeia of cholera which prescribes small doses (one or two grains) of calomel every five or ten minutes "until the symptoms of collapse become materially subdued."

Seventhly.—We have been told that neither in stimulation, calomelization, narcosis or venesection, is the cure for cholera to be found, but that it is in the free administration of vegetable and mineral acids, and of the sulphuric acid in particular, that the Το καλον lies embalmed.

Now, as neither of these "best plans" of treatment had their birth in the present epidemic, it was perhaps scarcely to be expected the pestilence should pass over without some warm and enthusiastic practitioner bringing another into the world. And so it has happened, and with a warmth of enthusiasm, too, beyond our expectation, considering the new method had only fifteen cases for its basis; and beyond our explanation, too, were it not that there is a fatality in the readiness with which physicians, who devote much time to physiologic and microscopic investigation, yield themselves up to strange vagaries of opinion on the nature and treatment of disease. Nevertheless, it is our duty to record in this place that at present the "method of treatment which," according to Dr. George Johnson, "promises a more than ordinary amount of success," is the administration of castor oil, "in doses of half an ounce every half-hour," and that "more than one patient has had as much as a pint in the course of forty-eight hours with impunity. Since this new method has been promulgated in the daily newspapers, there has been, as might be expected, a "run" upon castor oil, as there was upon oleum cajeputi (previously a cure for cholera) in 1831, when it rose from two to fourteen shillings an ounce. As in the case of the calomel, saline, and acid, &c., "plans," we receive a few communications in favor, but a multitude against this "new method."

Dr. Corner, of the Tower Hamlets Dispensary, writes to us to say that he has, "in several cases of very severe diarrhœa, given castor oil a fair trial, as recommended by Dr. Johnson, but it was only useful in mild cases, and in severe ones appeared to aggravate the symptoms; and that, in both diarrhœa and cholera, calomel, in from eight to fifteen grain doses, is the remedy which he has found most beneficial." Another correspondent informs us that those persons at Vera Cruz having its deadly fever, who are "treated by calomel, uniformly die," whilst those "treated by the administration of castor oil and lime juice, mixed," recover. He would therefore suggest the addition of lemon juice to the "new remedy" for cholera. A third, whose letter is before us, recommends "a cataplasm of mustard applied to the whole body, from the neck downwards;" while Mr. Chaplin, of the Bloomsbury Dispensary, informs us that, during the fortnight ending September 13th, "only four cases of *cholera* have presented themselves. These have all done well under the calomel, rhubarb, and saline plan of treatment." Our space will not permit us to give the details of Mr. Chaplin's communication, able though it be. In the September number of the *Pharmaceutical Journal* we find some observations bearing reference to our own remarks (quoted) of August the 19th, and pointing out the agents and formulæ druggists have been most called upon to dispense during the present epidemic. We extract the following:—" Sulphuric acid has of late been prominently brought forward. It is given in doses of twenty to forty minims, repeated every two or three hours, or oftener if occasion requires. Sometimes half a drachm or a drachm of chloric ether is added to each dose. Another prescription consists of twenty or thirty minims of laudanum, with about double the quantity of sulphuric ether; or the following:—Chloric ether, half a drachm; aromatic confection, one scruple; Battley's liquor opii, ten minims; spirit of sal volatile, half a drachm; mix."

We may also acknowledge the receipt of the third edition of Mr. Stephens's tract "On Cholera," in which it is taught that it is "to antiseptics we must look for the curative means" generally, and in particular to *creasote;* nor can we leave this department of the subject without recording the "important suggestion" (as he himself calls it) of another physiological physician, and its support by a chemical one, towards a satisfactory treatment of the stage of collapse. The former (B. W. Richardson, M. D.) advises, "that into the peritoneum or the cellular tissue of a patient in a proper state of collapse from cholera, water, to the extent of at least a tenth or even a fifth part of the whole weight of the body," be injected; the latter, (W. B. Herapath, M.D.,) believing this to be an "extremely philosophical and ingenious suggestion," thinks it might be improved—we mean the *water*—if its adviser would add *milk* to it, seeing we should thus "avoid the chances of destruction of the blood corpuscles," &c. We trust we may not be thought going beyond our proper duty in asking the calm attention of all past, present and future propounders of new methods of treating disease to the following quotation from the "Natural History" of Pliny, "Naturæ vero rerum vis atque majestas in omnibus momentis fide caret, si quis modo partes ejus ac non totam complectatur animo."—Lib. vii. c. 1.

Since the above was written, we have received very many other communications, sufficient, indeed, to supply matter for the next half-dozen numbers of the journal. Mr. Boddington describes more particularly the details connected with the administration of sulphuric acid, and Dr. Shorthouse gives us the results of his trial of Dr. Ayre's plan of treatment. Dr. Shorthouse believes it to be "a delusion and a humbug; in itself no doubt *harmless* enough, but, as it prevents other kinds of treatment being adopted, a great evil. . . . On examination after death, the calomel was found in the stomach unchanged. From the stomach of one man," he says, "I took *nine drachms* of calomel." In lieu of calomel," Dr. Shorthouse recommends the *phosphate of soda* as the agent *par excellence* in cholera. Mr. G. Selwyn Morris, on the contrary, writes to express his attachment to the doctrines of Dr. Ayre, and to recommend the addition of chloric ether to the medicines employed; whilst M. Meinig, in a communication, draws our attention to the value of continuous currents of electricity. Mr. Walker, of Burslem, favors us with his reasons for believing in the contagiousness of cholera, and that "there can remain but little room for doubt that its spread is generally to be attributed to that source;" while Dr. Robert Fowler, admitting it to be "communicable from one person to another,'

yet contends "that, in its *general diffusion*, it does not spread by contagion, but that the disease is a true epidemic, depending on telluric or atmospheric influences." Dr. Maxwell, amongst other items, furnishes us with two prescriptions, compounded of no less than *eighteen* ingredients, the oil of juniper and quinine appearing to us as the *pièces de resistance* in them.

A less complex therapeutic armatory is found in the new drug now being employed in Paris—viz., *guarana*, a substance procured from the seeds of Paullinia sorbilis, containing tannate of cafeine and a special resinous ingredient, &c.; and for drawing our attention to which we stand indebted to Mr. Howard Hopley.

We must close this notice by the acknowledgment of the receipt of Mr. Borham's "cases and remarks" on cholera, which, as he tells us, have been committed to paper, "not with any idea of being able to render things more clear with regard to its origin or pathology, nor to suggest anything particularly novel in its treatment," will scarcely demand more lengthened analysis. Mr. Borham's own opening luminous paragraph, indeed, forbids it. "At the mention," says he, "of the word Cholera, in its true sense, decimating as it is doing our population and army, the practical surgeon is struck with feelings of awe, and if he possess a mind, it becomes enveloped in a confusion of ideas which seem to get more confounded as he proceeds to think."

The result of the investigation by the Medical Council of the Board of Health into the treatment by *castor oil* has just been published. We have (at the last moment of going to press) only room for the following *resumé*:—

"It appears that in eighty-nine cases of cholera, treated by fourteen different practitioners with castor oil on the plan recommended by Dr. Johnson, sixty-eight were fatal, recovery having occurred only in fifteen cases, while the six remaining cases are still under treatment. The above report having been laid before the Council, and approved by them, it was resolved that the same be communicated to the President of the General Board of Health.

J. A. Paris,
B. G. Babington,
A. Tweedie,
W. Baly,
N. B. Ward.

"*Whitehall*, Sept. 15, 1854."

*London Lancet.*

